# A randomized controlled trial of sweet basil leaf powder‐enriched cookies for anemia management in adolescent girls

**DOI:** 10.1002/fsn3.4098

**Published:** 2024-03-15

**Authors:** Farah Naz Akbar, Shahid Mahmood, Ghulam Mueen‐ud‐din, Waseem Khalid, Muhammad Zubair Khalid, Zaira Aziz, Saleh Alfarraj, Mohammad Javed Ansari, Felix Kwashie Madilo

**Affiliations:** ^1^ Institute of Food Science and Nutrition University of Sargodha Sargodha Pakistan; ^2^ Department of AHS Sargodha Medical College, University of Sargodha Sargodha Pakistan; ^3^ University Institute of Food Science and Technology, The University of Lahore Lahore Pakistan; ^4^ Department of Food Science Government College University Faisalabad Faisalabad Pakistan; ^5^ Pakistan Institute of Medical Sciences Islamabad Pakistan; ^6^ Zoology Department College of Science, King Saud University Riyadh Saudi Arabia; ^7^ Department of Botany Hindu College Moradabad (Mahatma Jyotiba Phule Rohilkhand University Bareilly) Moradabad India; ^8^ Department of Food and Science and Technology Ho Technical University Ho Ghana

**Keywords:** adolescent girls, anemia, Hb level, hematological parameters, nutritional status

## Abstract

The study aimed to evaluate the effectiveness of sweet basil leaf powder as a natural source of iron for the treatment of anemia in adolescent girls. Purposive sampling technique of two‐stage sampling; part of the nonprobability sampling approach. Out of 2400 approached adolescent girls, 1645 agreed to participate and their nutritional status was assessed. Of these, 89.95% had clinical signs and symptoms of anemia, and 59.79% were found to be anemic based on Hb levels. From the anemic group, 65.18% were randomly selected to receive either B_0_ (Control), B1 (12.699 g FeSO_4_.7H_2_O/100 g), and B3 (16 g SBLP/100 g) cookies for 4 months. At the end of the intervention, the assessment of nutritional status, complete blood count, serum iron, serum ferritin, serum total iron‐binding capacity (TIBC), and transferrin saturation was explored. Hematological parameters such as Hb, Hct, TIBC, MCV, MCH, MCHC, serum iron, and serum ferritin were significant (*p* ≤ .05). The result showed that the serum Fe was highest in group B_3_ while a significant decline was noted for group B_0_. Serum ferritin for B_1_ was better than B_3_. The entire treatment for transferrin saturation showed a highly significant increasing trend in B_3_ and B_1_, regardless of the control. TIBC levels raised in the control group while in all other treatments, it declined. The study demonstrated that SBLP‐fortified cookies can be an effective treatment option for anemia, as evidenced by significant improvements in key hematological parameters.

## Graphical abstract

1. The effectiveness of sweet basil leaf powder as a natural source of iron for the treatment of anemia in adolescent girls.
2. Hematological parameters such as Hb, Hct, TIBC, MCV, MCH, MCHC, serum iron, and serum ferritin were significant (*p* ≤ .05).
3. The study demonstrated that SBLP‐fortified cookies can be an effective treatment option for anemia.

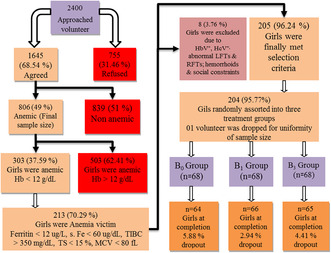

## 1 INTRODUCTION

Iron, a vital micronutrient, plays a central role in maintaining human health and well‐being. Adequate iron intake is crucial for various physiological functions, including oxygen transport, energy production, and immune system maintenance. Anemia is prevalent in adolescent girls, characterized by insufficient red blood cells or hemoglobin to transport oxygen to the body's tissues. Iron deficiency is the most common cause, resulting from factors like poor diet, heavy menstrual bleeding, and rapid growth during puberty. Public health programs have historically neglected this vulnerable period in human life, with girls being more likely to become anemic due to limited resources, lack of good food and education, and extra household work. Signs of anemia in adolescent girls may include weakness, reduced appetite, fatigue, dizziness, headaches, pale skin, and shortness of breath during exercise. In this context, the consumption of plant‐based foods rich in iron holds paramount importance for ensuring optimal iron nutrition, particularly within the framework of balanced diets and sustainable food systems. Plant‐based foods offer a valuable solution to iron deficiency due to their inherent iron content, as well as the presence of other nutrients that enhance iron absorption. While animal sources like red meat are traditionally associated with high iron content, various plant‐based foods can provide a substantial amount of this essential mineral (Wang & Scrimgeour, 2021). These foods not only supply iron but also contribute to improved iron absorption. Beyond their nutritional benefits, these foods align with the principles of sustainability and environmental conservation. The production of plant‐based foods generally requires fewer resources, including water and land, compared to animal agriculture. As the world grapples with the challenges of climate change and resource scarcity, transitioning toward diets rich in plant‐based foods becomes a pivotal step toward a more sustainable future. While it also offers a promising solution to iron deficiency, it is important to acknowledge potential challenges. Treatment involves a diet rich in iron‐containing foods, fortification of staple foods, and the use of medicinal plants rich in minerals. Sweet Basil (*Ocimum basilicum* L.) is a nutrient‐rich herb with several health benefits, including vitamins and minerals such as vitamin C, calcium, and potassium (Şen et al., 2019). Mostly fresh leaves are used but dry leaves are rich source of nutrients as compared to the fresh. Its components have traditionally been used to flavor food, dental care, and oral drugs (Singletary, 2018). Iranian basils are used for managing fevers, inflammation of the throat, and stomachache (Babaeian et al., 2015; Kulkarni et al., 2012). According to El Khouly (2017), antianemic activity of sweet basil remains unknown although it showed high content of Fe (89.80 mg/100 g). Incorporating dried basil leaves into cookies can be a practical way to address nutrient deficiencies, specifically in adolescent girls. A study was designed to evaluate the effectiveness of sweet basil leaf powder as a natural source of iron for the treatment of anemia in adolescent girls. SBLP (Fe)‐fortified cookies have been administered in adolescent girls to overcome anemia. It has been a very slow yet effective process to control anemia in young girls. Therefore, it is also a better and cost‐effective option to utilize sweet basil (SB) in cookies through the fortification process for meeting the partial requirement of iron, especially in adolescents.

## 2 MATERIALS AND METHODS

Approval for the research work was granted by the Biosafety and Ethical Review Committee at the University of Sargodha, Sargodha, Pakistan.

### 2.1 Procurement of raw materials

Five different treatments of cookies using varying amounts of SBLP, that is, Treatment (B_1_) includes 12.699 mg of FeSO_4_.7H_2_O with EDTA, excluding SBLP. Treatments (B_2_), (B_3_), and (B_4_) incorporate SBLP at 14, 16, and 18 g, respectively, without adding FeSO_4_.7H_2_O. Treatment (B_0_) involves no specific additions of either agent and was prepared according to the method listed in AACC (2000) with some modifications, sealed in wraps of bi‐oriented polypropylene (BOPP), packed in containers, and stored at room temperature.

### 2.2 Experimental design and data collection

**FIGURE 1:**
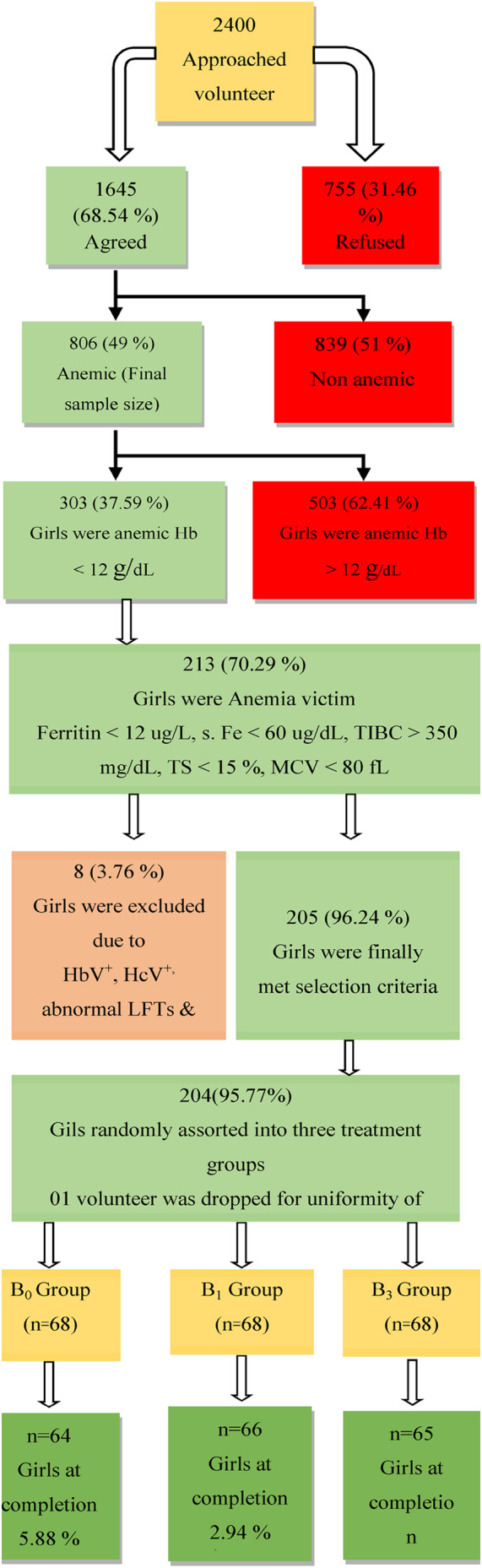
Assortment of anemic adolescent girls.

For the study, multiple schools and colleges within the Sargodha region were selected as study sites. To collect data from adolescent girls, a two‐stage sampling technique was used, which is classified as a nonprobability sampling approach according to Muhammad (2000). One from the best treatment; SBLP, that is, B_3_, Ferrous sulfate, that is, B_1_, and along with B_0_ (control) were selected based on physicochemical, sensory, and stability analysis results as shown in Figure 1. Considering the RDA of iron (13.6 mg) for anemic adolescent girls, fortified cookies were prepared. Of those who met the inclusion criteria, 38.83% (*n* = 313) had Ferritin levels < 12 μg/L, s. Fe < 60 μg/dL, TIBC > 350 mg/dL, TS < 15%, MCV < 80 fL. Out of these, 108 were excluded from the study due to hepatitis B and C, renal anomalies, and hemorrhoids. Finally, 204 volunteers were randomly selected into three treatment groups, B_0_ (control/placebo group), B_1_ (FeSO_4_.7H_2_O group), and B_3_ (SBLP group), with 68 volunteers in each group.

Each group was provided with three different iron treatments for 120 days to explore their impacts. After the preliminary trial, best cookie treatments, that is, B_0_ (Control), B_1_ (12.699 FeSO_4_.7H_2_O/100 g), and B_3_ (16 g SBLP/100 g) were selected for efficacy studies to furnish 50% of RDA of Fe (13.6 mg). The selected cookie treatments were prepared in the lab of IFSN University of Sargodha, with five cookies (50 g) providing 50% RDA of anemic adolescent girls (7 mg). Cookies were provided to schools/colleges on a weekly basis for distribution to the target girls.

### 2.3 Assessment of demographics and anthropometrics of anemic adolescent girls

Demographic details such as the name of the volunteer, ethnicity, religion, marital status, education, and socioeconomics were collected. The girl's body measurements like height (Gordon, 1983), weight, composition of the body, and body mass index (BMI) were recorded (Baumgartner et al., 1998).

### 2.4 Anemia assay biomarkers

Volunteers' blood was obtained and tested to hematological parameters such as serum iron, serum ferritin, Hb, MCV, MCH, and TIBC and identifying anemia (Gibney et al., 2008).

### 2.5 Assessment of dietary intakes

Each of the baseline volunteers filled proforma of dietary consumption data to compare the food diary at the baseline and completion of study (Rockett et al., 1995). A diet plan was also prepared for anemic adolescent girls focusing on incorporating iron‐rich foods into their meals and snacks, along with foods that help to increase the absorption of iron and better their eating habits (Table 1). It also included fortified cookies which were approximately 2100–2650 calories.

**TABLE 1:** Diet plan with iron‐rich food.

Time	Food	Kcal
Breakfast	Whole grain bread/paratha with scrambled eggs	200–250
	Milk/milkshakes	100–150
	Almond butter	100–150
Snack	Iron‐fortified cookies with orange/strawberries juice (the vitamin C in the orange/strawberries can help increase iron absorption)	400–450
Lunch	Grilled chicken breast with whole wheat bread	300–350
	Lettuce and tomato salad	150–200
	Sweet potato fries (sweet potatoes are a good source of iron)	150–200
Snack	Apple slices with almond butter (almonds are a good source of iron)	150–200
Dinner	Rice with roasted chicken and yogurt	350–450
	Mixed green salad with sliced red bell pepper and a vinaigrette dressing	150–200
Snack	Hummus with carrot sticks (A good source of iron)	150–200

### 2.6 Statistical analysis

Quantitative data were collected according to factorial layout under CRD and analyzed using SPSS. Variance analysis (ANOVA) methodology (Steel et al., 1997) was used to analyze the data, and the Tukey's test was used to evaluate significance among groups (Abdi et al., 2009). The significance level was set at *p* ≤ .05. Descriptive statistics were used to evaluate the distribution and frequency of qualitative data, which helped to reach the inferences and conclusions.

## 3 RESULTS

All participants were between the ages of 13 and 19. The largest ethnic group was Punjabi, accounting for 139 volunteers (71.3%), followed by Saraiki with 49 volunteers (25.1%). The religious affiliation of the volunteers consisted of 186 Muslims (95.4%), 1 Christian (1.5%), and 8 Qadiani/Ahmadi (4.1%). In terms of education, 115 volunteers (59%) were pursuing graduation, 60 (30.8%) were pursuing intermediate studies, 14 (7.2%) were pursuing matriculation, and 6 (3.1%) were pursuing other courses. According to the marital status, 193 volunteers (99%) were unmarried while only two (1%) were married, as evident from the frequency distribution table (Table 2). In terms of income, the frequency distribution table shows that 89 volunteers (45.6%) belonged to the low‐middle class, 103 (52.8%) were from the middle‐income bracket, while three (1.5%) had income above PKR ≥ 2,071,992.

**TABLE 2:** Demographic distribution of volunteers.

Variables	Response	Frequency (ƒ)	Percentage (%)
Ethnicity	Punjabi	139	71.3
	Balochi	0	0
	Sindhi	0	0
	Pathan	7	3.6
	Kashmiri	0	0
	Gilgiti	0	0
	Saraiki	49	25.1
	Others	0	0
Religion	Islam	186	95.4
	Christian	1	0.5
	Qadiani/Ahmadi	8	4.1
Marital status	Married	2	1.0
	Unmarried	193	99.0
Education	Graduation	115	59.0
	Intermediate	60	30.8
	Matric	14	7.2
	Others	6	3.1
Socioeconomics	Lower middle income (PKR 171,235–668,480)	89	45.6
	Middle income (PKR 668,719–2,071,545)	103	52.8
	High income (PKR ≥ 2,071,992)	3	1.5

According to Table 3, the anthropometrics of volunteers in all treatment groups were 16.797 ± 0.2043, 16.831 ± 0.1925, and 16.831 ± 0.1968 for B_0_, B_1_, and B_3_, respectively. There was no significant relationship observed among adolescent girls in terms of their body weight (46.190 ± 1.3076 to 45.624 ± 1.6150 kg), height (154.35 ± 1.0651 to 155.81 ± 1.0421 cm), and BMI (19.25 ± 0.4128 to 19.26 ± 0.4116 kg/m^2^) at both the baseline and end of the study.

**TABLE 3:** Impact of treatments on the anthropometrics of anemic adolescent girls.

Variable	Groups	Days		Means ± *SEM*
		0	120	
Weight (kg)	B_0_	46.190 ± 1.307	45.624 ± 1.615	55.907 ± 1.043
	B_1_	46.026 ± 1.143	46.689 ± 1.186	56.358 ± 0.820
	B_3_	46.005 ± 1.214	46.739 ± 1.218	56.372 ± 0.858
	Means ± *SEM*	46.074 ± 0.701	46.350 ± 0.781	
Height (cm)	B_0_	154.35 ± 1.065	154.36 ± 1.065	154.35 ± 0.856
	B_1_	155.81 ± 1.042	156.82 ± 1.041	155.91 ± 0.734
	B_3_	155.75 ± 1.043	155.76 ± 1.046	155.83 ± 0.739
	Means ± *SEM*	155.30 ± 0.635	155.30 ± 0.638	
BMI (kg/m^2^)	B_0_	19.81 ± 0.4828	19.58 ± 0.5198	19.70 ± 0.357
	B_1_	19.41 ± 0.3947	19.43 ± 0.3751	19.42 ± 0.2713
	B_3_	19.25 ± 0.4128	19.26 ± 0.4116	19.26 ± 0.290
	Means ± *SEM*	19.33 ± 0.2482	19.42 ± 0.2540	

*Note*: All means in the table are non‐significant at (*p* > .05).

Table 4 presents the impact of different treatments on the body composition of anemic adolescent girls including body water, fat, muscle mass, and bone mass among all three groups, namely, B_0_ (control group), B_1_ (FeSO_4_.7H_2_O group), and B_3_ (SBLP group), with measurements taken at two time points, 0 and 120 days. For body water, the mean percentages were similar across the groups at day 0, with B_0_ at 50.018%, B_1_ at 50.265%, and B_3_ at 50.239%. At day 120, the mean percentage of body water was highest in the B_3_ group at 51.072%, followed by B_1_ at 50.481% and B_0_ at 49.126%. The changes in body water between days 0 and 120 were not large for any of the groups. For fat, the mean percentages were similar across the groups at both time points. At day 0, the mean percentages were B_0_ at 21.74%, B_1_ at 21.81%, and B_3_ at 21.32%. At day 120, the mean percentages were B_0_ at 21.96%, B_1_ at 21.79%, and B_3_ at 21.59%. For muscle mass, the mean percentages were similar across the groups at day 0, with B_0_ at 30.296%, B_1_ at 30.467%, and B_3_ at 30.003%. At day 120, the mean percentage of muscle mass was highest in the B_3_ group at 31.000%, followed by B_1_ at 30.747% and B_0_ at 29.187%. The changes in muscle mass between days 0 and 120 were small for B_0_ and B_1_, but larger for B_3_. For bone mass, the mean values were similar across the groups at both time points. At day 0, the mean values were B_0_ at 4.160 kg, B_1_ at 4.135 kg, and B_3_ at 4.133 kg. At day 120, the mean values were B_0_ at 4.102 kg, B_1_ at 4.200 kg, and B_3_ at 4.9635 kg. The changes in bone mass between day 0 and 120 were small for B_0_ and B_1_, but larger for B_3_. The results suggest that the SBLP treatment (B_3_) may have a positive impact on muscle and bone mass among anemic adolescent girls, while the FeSO_4_.7H_2_O treatment (B_1_) did not show significant differences compared to the control group (B_0_) for any of the variables measured.

**TABLE 4:** Impact of treatments on body composition of anemic adolescent girls.

Variable	Groups	Days		Means ± *SEM*
		0	120	
Body water (%)	B_0_	50.018 ± 0.8618	49.126 ± 0.8618	49.572 ± 1.234
	B_1_	50.265 ± 0.5990	50.481 ± 0.5990	50.373 ± 0.439
	B_3_	50.239 ± 0.6357	51.072 ± 0.6357	50.656 ± 0.461
	Means ± *SEM*	50.174 ± 0.3798	50.226 ± 0.9071	
Fat (%)	B_0_	21.74 ± 0.6315	21.96 ± 0.6315	21.85 ± 1.545
	B_1_	21.81 ± 4.8384	21.79 ± 4.8384	21.80 ± 2.454
	B_3_	21.32 ± 4.0514	21.59 ± 4.0514	21.44 ± 2.853
	Means ± *SEM*	21.63 ± 1.6848	21.78 ± 2.1497	
Muscle mass (%)	B_0_	30.29 ± 0.6957	29.187 ± 0.6957	29.741 ± 0.739
	B_1_	30.46 ± 0.2798	30.747 ± 0.2798	30.607 ± 0.196
	B_3_	30.01 ± 0.2828	31.000 ± 0.2828	30.502 ± 0.199
	Means ± *SEM*	30.34 ± 0.1637	30.218 ± 0.5469	
Bone mass (kg)	B_0_	4.16 ± 0.1291	4.102 ± 0.1291	4.131 ± 0.092
	B_1_	4.13 ± 0.1157	4.200 ± 0.1157	4.1675 ± 0.080
	B_3_	4.13 ± 0.1197	4.194 ± 0.1197	4.9635 ± 0.083
	Means ± *SEM*	4.14 ± 0.0697	4.165 ± 0.0699	

*Note*: All means in the table are non‐significant at (*p* > .05).

It was revealed from Table 5 that the iron‐fortified treatment groups (B_1_, ferrous sulfate‐fortified group and B_3_, SBLP‐fortified group) showed a significant increase in red blood cell (RBC) levels. The mean RBC level increased from 25.333 ± 0.0503 to 41.511 ± 0.0327 g/dL in group B_3_, while the mean RBC level decreased from 25.455 ± 0.0344 to 24.053 ± 0.1219 g/dL in the control group (B_0_). The study also showed that the serum ferritin levels were highest in the B_1_ group (10.827 ± 0.1921 to 20.611 ± 0.6079 ng/mL) compared to B_3_ (10.631 ± 0.0471 to 20.016 ± 0.1033 ng/mL) and the control group. Total iron binding capacity (TIBC) levels decreased the most in B_3_ (390.71 ± 0.31 to 376.61 ± 0.61 μg/dL) and B_1_ (390.44 ± 0.82 to 374.84 ± 0.59 μg/dL). The final mean of transferrin saturation (%) in B_1_ was 13.016 ± 0.0983%, in B_3_ it was 11.039 ± 0.0434%, and in B_0_ it was 10.566 ± 0.0953%. The Hb level increased gradually in both treatment groups fortified by iron B_1_, that is, ferrous sulfate, B_3_, that is, SBLP and decreased in control group, that is, B_0_ at the end of 4‐month study. The mean values of Hb for B_0_ were 9.252 ± 0.0503 g/dL at baseline and 7.234 ± 0.0327 g/dL at the end of the study. For B_1_, the mean values were 9.416 ± 0.0503 g/dL at baseline and 12.513 ± 0.0327 g/dL at the end of the study. For B_3_, the mean values were 9.438 ± 0.0503 g/dL at baseline and 14.786 ± 0.0327 g/dL at the end of the study. The means of Hct indices in control group, that is, B_0_ were significantly reduced from 31.269 ± 0.0446 to 30.946 ± 0.1449% while highly significant improvement from 31.365 ± 0.0333 to 33.314 ± 0.2151% in B_1_ group and 31.250 ± 0.0211 to 33.590 ± 0.1819% in B_3_ was observed. Treatment B_3_ of SBLP‐fortified group (77.064 ± 0.2325 to 78.408 ± 0.2100 fL) was better than B_1_ (ferrous sulfate‐fortified group). In the control group, a highly significant drop (27.173 ± 0.0956 to 26.916 ± 0.1238 pg) was noted and improvement (27.545 ± 0.1956 to 28.688 ± 0.0592 pg) was observed in B_3_. The highest rise was (35.522 ± 0.0552 to 33.856 ± 0.0397 g/dL) in B_3_ group and B_0_ group showed a drop (33.448 ± 0.1203 to 29.692 ± 0.6239 g/dL) that differs significantly from other treatments. Overall, the study demonstrated that SBLP‐fortified treatments had a positive effect on anemic adolescent girls.

**TABLE 5:** Impact of treatments on the biomarkers of anemic adolescent girls.

Variable	Groups	Days		Means ± *SEM*
		0	120	
Serum iron (g/dL)	B_0_	25.455 ± 0.0344^c^	24.053 ± 0.1219^d^	24.754 ± 0.0876^C^
	B_1_	25.482 ± 0.0355^c^	39.938 ± 0.3382^b^	32.710 ± 0.6544^B^
	B_3_	25.333 ± 0.0503^c^	41.511 ± 0.0327^a^	33.422 ± 0.7184^A^
	Means ± *SEM*	25.423 ± 0.0237^B^	35.167 ± 0.5766^A^	
Serum ferritin (ng/mL)	B_0_	11.637 ± 0.0233^c^	10.600 ± 0.2123^c^	11.119 ± 0.3454^C^
	B_1_	10.827 ± 0.1921^c^	13.611 ± 0.6079^b^	12.219 ± 0.5489^B^
	B_3_	10.631 ± 0.0471^c^	14.016 ± 0.1033^a^	12.324 ± 0.5080^A^
	Means ± *SEM*	11.032 ± 0.0728^B^	12.740 ± 0.6750^A^	
Transferrin saturation (%)	B_0_	11.006 ± 0.0298^c^	10.566 ± 0.0953^c^	10.7858 ± 0.0528^C^
	B_1_	11.014 ± 0.0330^c^	12.737 ± 0.2381^b^	11.8756 ± 0.2030^B^
	B_3_	11.039 ± 0.0434^c^	13.016 ± 0.0983^a^	12.0280 ± 0.2272^A^
	Means ± *SEM*	11.019 ± 0.0205^B^	12.110 ± 1.3902^A^	
Total iron‐binding capacity [TIBC] (μg/dL)	B_0_	391.23 ± 1.41^b^	405.41 ± 0.62^a^	398.32 ± 0.06^A^
	B_1_	390.71 ± 0.31^b^	376.61 ± 0.61^c^	383.66 ± 1.08^B^
	B_3_	390.44 ± 0.82^b^	374.84 ± 0.59^c^	382.64 ± 1.15^B^
	Means ± *SEM*	390.79 ± 0.407^A^	385.62 ± 1.03^B^	
Red blood cells [RBCs] (M/μL)	B_0_	4.1076 ± 0.0116^b^	4.0367 ± 0.0142^c^	4.0721 ± 0.0116^B^
	B_1_	4.1046 ± 0.0139^b^	4.2954 ± 0.0132^a^	4.1998 ± 0.0139^A^
	B_3_	4.1172 ± 0.0314^b^	4.3281 ± 0.0102^a^	4.2226 ± 0.0314^A^
	Means ± *SEM*	4.1098 ± 0.0110^B^	4.220 ± 8.27E‐03^A^	
Hb (g/dL)	B_0_	11.377 ± 0.0164^c^	10.405 ± 0.0427^d^	10.891 ± 0.0483^C^
	B_1_	11.402 ± 0.0394^c^	12.452 ± 0.0369^b^	11.927 ± 0.0531^B^
	B_3_	11.356 ± 0.0409^c^	12.705 ± 0.0182^a^	12.030 ± 0.0638^A^
	Means ± *SEM*	11.379 ± 0.0195^B^	11.854 ± 0.0761^A^	
Hct (%)	B_0_	31.269 ± 0.0446^b^	30.946 ± 0.1449^b^	31.107 ± 0.0758^B^
	B_1_	31.365 ± 0.0333^b^	33.314 ± 0.2151^a^	32.340 ± 0.1391^A^
	B_3_	31.250 ± 0.0211^b^	33.590 ± 0.1819^a^	32.420 ± 0.1382^A^
	Means ± *SEM*	31.295 ± 0.0202^B^	32.617 ± 0.1355^A^	
MCV (fL)	B_0_	77.005 ± 0.3141^b^	75.008 ± 0.3883^c^	76.736 ± 0.3119^B^
	B_1_	77.047 ± 0.2644^b^	78.233 ± 0.2488^a^	77.640 ± 0.1872^A^
	B_3_	77.064 ± 0.2325^b^	78.408 ± 0.2100^a^	77.736 ± 0.1581^A^
	Means ± *SEM*	77.039 ± 0.1584^B^	77.243 ± 0.2501^A^	
MCH (pg)	B_0_	27.173 ± 0.0956^bc^	26.916 ± 0.1238^c^	27.044 ± 0.0784^B^
	B_1_	27.217 ± 0.0934^bc^	28.664 ± 0.0851^a^	27.941 ± 0.0891^A^
	B_3_	27.545 ± 0.1956^b^	28.688 ± 0.0592^a^	28.116 ± 0.1137^A^
	Means ± *SEM*	27.312 ± 0.0788^B^	28.089 ± 0.0798^A^	
MCHC (g/dL)	B_0_	33.448 ± 0.1203^b^	29.692 ± 0.6239^c^	31.570 ± 0.3536^B^
	B_1_	33.532 ± 0.1239^b^	35.307 ± 0.1255^a^	34.330 ± 0.1221^A^
	B_3_	33.856 ± 0.0397^b^	35.522 ± 0.0552^a^	34.698 ± 0.0820^A^
	Means ± *SEM*	33.559 ± 0.0610	33.507 ± 0.2866	

*Note*: Capital letter superscripts show the statistically significant differences of means within the same treatment group and small letter superscripts show the statistically significant differences of means among the different treatment groups.

**FIGURE 2:**
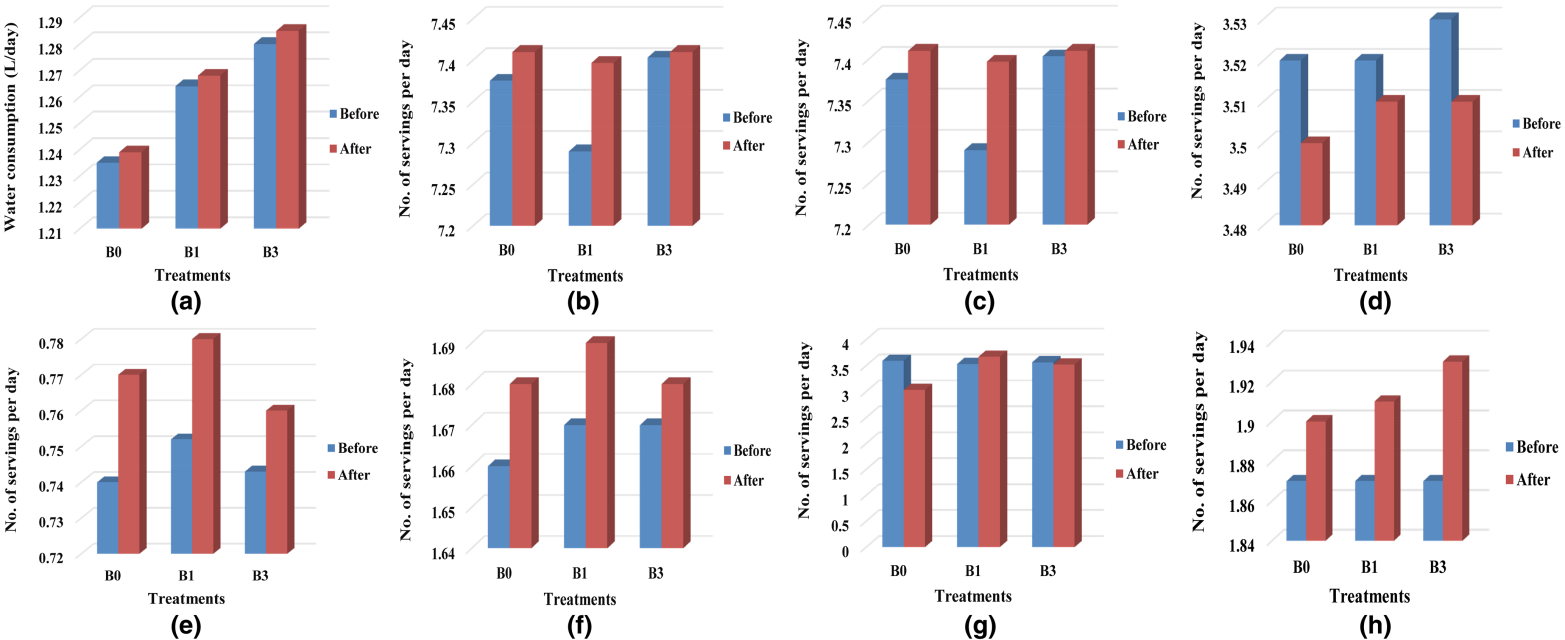
Consumption of different food groups among adolescent girls: (a) Consumption of water, (b) Consumption of cereal, bread, rice and pasta, (c) Consumption of vegetable, (d) Consumption of fats, oils and sweets, (e) Consumption of fruit, (f) Consumption of milk, yoghurt and cheese, (g) Consumption of junk food, (h) Consumption of meat, fish, poultry, beans, eggs and nuts.

Figure 2a‐h presents information on the mean values of various dietary variables for three different groups (B_0_, B_1_, and B_3_) before and after a study. The variables included water consumption and servings of different food groups (cereal, bread, rice and pasta, vegetables, fats/oils/sweets, fruits, milk/yogurt/cheese, junk food, and meat/fish/poultry/beans/eggs/nuts). By comparing the means before and after the study, one could evaluate the change in dietary behavior among the different groups. It was observed that the mean water consumption increased for all three groups after the study. Similarly, the mean servings of cereal, bread, rice, and pasta decreased for groups B_1_ and B_3_ after the study compared to before the study. The mean servings of vegetables increased for all three groups after the study. The changes observed in the eating behavior of the girls before and after this study were due to the diet plan and personal motivation that was given to them during the study. The diet plan included different types of iron‐rich foods and iron‐fortified cookies which may have influenced the girls' iron deficiency as well as the diet plan may have been designed to improve the girls' iron deficiency, overall health, and wellness, which could have led to positive changes in their eating habits.

## 4 DISCUSSION

The results of the study provided demographic information about the participants, such as their age, ethnicity, religion, education, marital status, and income. The majority of the participants were Punjabi, Muslim, and pursuing higher education. This demographic profile is consistent with Pries et al. (2019) studies conducted in Pakistan, which showed that the majority of the population is Muslim and that education is highly valued. However, the study did not provide any analysis of how these demographic factors may have influenced the results of the study. The results of this study indicate that there was no significant difference in anthropometric measurements (body weight, height, and BMI) among the three treatment groups at the beginning and end of the study. This finding was consistent with some studies that have found no significant relationship between iron supplementation and anthropometric measurements in adolescent girls (Ahmad et al., 2015; Salam et al., 2016).

However, there are also studies that have reported a positive effect of iron supplementation on anthropometric measurements in adolescent girls. For example, a study conducted by Dad et al. (2017) in Pakistan found that iron supplementation for 6 months resulted in significant improvements in weight and height of adolescent girls. It is important to note that the duration and dosage of iron supplementation may play a role in the effect on anthropometric measurements. Dostal et al. (2014) mentioned that the duration of iron supplementation was 8 weeks, which may not have been sufficient to observe significant changes in anthropometric measurements.

The results of this study were consistent with previous research demonstrating that iron‐fortified treatments can effectively improve RBC levels and increase serum ferritin levels in anemic individuals. In a study conducted by Glinz et al. (2017), it was found that iron‐fortified foods increased RBC levels and serum ferritin levels in women with iron deficiency anemia. Another study by Kumar et al. (2022) revealed that iron‐fortified biscuits improved the hemoglobin levels in anemic adolescent girls.

The results of this study indicate that fortified food with SBLP and ferrous sulfate had a significant positive effect on the hemoglobin, hematocrit, and serum iron levels of anemic adolescent girls compared to the control group. These findings are consistent with the results of several other studies that have shown that iron‐fortified foods can improve the iron status of anemic individuals. A study conducted by Hess et al. (2016) showed that iron‐fortified soy sauce had a significant positive effect on the iron status of anemic women in China. Kuong et al. (2016) demonstrated that iron‐fortified rice was effective in improving the iron status of anemic women in Cambodia. Similarly, in a study conducted by Abdalla (2018), it was found that iron‐fortified bread had a positive effect on the iron status of anemic women in Korea.

The current study also found that SBLP‐fortified food had a similar effect on the iron status of anemic adolescent girls as ferrous sulfate‐fortified food. This is consistent with the findings of a study conducted by Manggul et al. (2021), which showed that iron‐fortified biscuits containing Moringa oleifera leaves had a similar effect on the hemoglobin levels of anemic adolescent girls as ferrous sulfate‐fortified biscuits. Present study analyzed changes in dietary behavior among three groups (B_0_, B_1_, and B_3_) before and after the study, including water consumption and servings of different food groups. Mean water consumption increased for all three groups, while mean servings of cereal, bread, rice, and pasta decreased for groups B_1_ and B_3_, and mean servings of vegetables increased for all three groups. The changes were attributed to a diet plan and personal motivation, including iron‐rich foods and iron‐fortified cookies, designed to improve the girls' iron deficiency and overall health and wellness, leading to positive changes in their eating habits. A similar finding by Association (2007) showed that 91% of college students had expectations that they had good health status but their total consumption was 7%. In the present situation, fewer than 10% of U.S. college students say that they eat five required regular portions of fruits and vegetables. Croezen et al. (2009) recorded that the rise of unhealthy eating habits and the growing consumption of sweetened drinks by young people played an important role in obese and atherosclerosis children. In a similar study by Collison et al. (2010), it was shown that current malnutrition is the result of growing unhealthy eating habits among women. Younger part of a population often stays away from breakfast which adds to the risk of being either undernourished or obese. However, increasing water intake and consumption of fruits, vegetables, and whole grains are recommended as part of a healthy diet. Additionally, limiting consumption of foods high in added sugars, saturated fats, and sodium is recommended to improve overall health outcomes.

## 5 CONCLUSION

In conclusion, all treatment groups showed significant changes in anemia indices, while safety indices remained normal. The group that received 16% SBLP (B_3_) demonstrated the highest increase in Hb (11.8%), serum Fe (63.88%), serum ferritin (31.84%), and transferrin saturation (17.91%), along with a decrease in TIBC (4.0%) while reverse of all was explored in B_0_ (control group). the findings of this study support the use of iron‐fortified foods, including those fortified with plant‐based sources of iron like SBLP, as a strategy to improve the iron status of anemic adolescent girls. Additionally, the implementation of a diet plan and personal motivation, which included iron‐rich foods and iron‐fortified cookies, was successful in improving the iron deficiency and overall health and wellness of adolescent girls and also led to positive changes in their eating habits.

## AUTHOR CONTRIBUTIONS

**Felix Kwashie Madilo:** Data curation (equal); formal analysis (equal); methodology (equal); writing – review and editing (equal). **Farah Naz Akbar:** Conceptualization (equal); data curation (equal); investigation (equal). **Shahid Mahmood:** Data curation (equal); formal analysis (equal); funding acquisition (equal); investigation (equal). **Ghulam Mueen‐ud‐din:** Conceptualization (equal); formal analysis (equal); software (equal); writing – original draft (equal). **Waseem Khalid:** Software (equal); supervision (equal); writing – original draft (equal); writing – review and editing (equal). **Muhammad Zubair Khalid:** Conceptualization (equal); data curation (equal); investigation (equal); software (equal); supervision (equal); writing – original draft (equal); writing – review and editing (equal). **Zaira Aziz:** Formal analysis (equal); funding acquisition (equal); investigation (equal); validation (equal); writing – original draft (equal). **Saleh Alfarraj:** Data curation (equal); investigation (equal); writing and editing (equal); writing – original draft (equal); funding acquisition (equal). **Mohammad Javed Ansari:** Formal analysis (equal); funding acquisition (equal); investigation (equal); writing – review and editing (equal).

## FUNDING INFORMATION

Not applicable.

## CONFLICT OF INTEREST STATEMENT

No potential conflict of interest declared by the author(s).

## Data Availability

The data collected for this research are included in the manuscript.
